# Dynamic risk control by human nucleus accumbens

**DOI:** 10.1093/brain/awv285

**Published:** 2015-10-01

**Authors:** Parashkev Nachev, Fernando Lopez-Sosa, Javier Jesus Gonzalez-Rosa, Ana Galarza, Josue Avecillas, Jose Angel Pineda-Pardo, Juan José Lopez-Ibor, Blanca Reneses, Juan Antonio Barcia, Bryan Strange

**Affiliations:** 1 Institute of Neurology, UCL, London, UK; 2 Laboratory for Clinical Neuroscience, Centre for Biomedical Technology, Technical University of Madrid, Spain; 3 Department of Neurosurgery, Hospital Clínico San Carlos, Instituto de Investigación Sanitaria San Carlos, Universidad Complutense de Madrid, Madrid, Spain; 4 CINAC, HM Puerta del Sur, Hospitales de Madrid, Móstoles, and CEU-San Pablo University, Madrid, Spain; 5 Department of Psychiatry, Hospital Clínico San Carlos, Instituto de Investigación Sanitaria San Carlos, Universidad Complutense de Madrid, Madrid, Spain

**Keywords:** nucleus accumbens, risk, reward, decision-making, subcortical electrical stimulation

## Abstract

Real-world decisions about reward often involve a complex counterbalance of risk and value. Although the nucleus accumbens has been implicated in the underlying neural substrate, its criticality to human behaviour remains an open question, best addressed with interventional methodology that probes the behavioural consequences of focal neural modulation. Combining a psychometric index of risky decision-making with transient electrical modulation of the nucleus accumbens, here we reveal profound, highly dynamic alteration of the relation between probability of reward and choice during therapeutic deep brain stimulation in four patients with treatment-resistant psychiatric disease. Short-lived phasic electrical stimulation of the region of the nucleus accumbens dynamically altered risk behaviour, transiently shifting the psychometric function towards more risky decisions only for the duration of stimulation. A critical, on-line role of human nucleus accumbens in dynamic risk control is thereby established.

## Abstract (precis)

The nucleus accumbens is a key node in the network linking reward to action. Studying a rare series of patients with bilaterally implanted electrodes in the nucleus accumbens, Nachev *et al.* show that external electrical stimulation of the accumbens dynamically shifts behaviour towards more risky decision making.

## Graphical abstract

**Figure:**
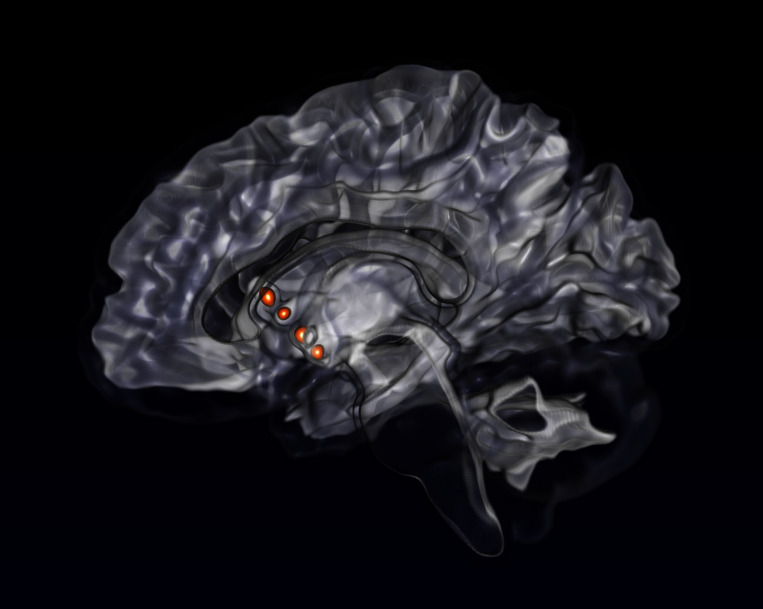
The nucleus accumbens is a key node in the network linking reward to action. Studying a rare series of patients with bilaterally implanted electrodes in the nucleus accumbens, Nachev *et al.* show that external electrical stimulation of the accumbens dynamically shifts behaviour towards more risky decision making.

## Introduction

A cardinal problem in the neuroscience of human behaviour is the nature of the mediating link between a potential reward and the action intended to secure it (Platt and Huettel, 2008; Rangel *et al.*, 2008). Within the subcortex, human structural and functional imaging data have implicated the nucleus accumbens, a subregion of the ventral striatum exhibiting patterns of neuroanatomical connectivity and task-related neural activity optimally suited to such a role (Schultz *et al.*, 1997; Matthews *et al.*, 2004; O’Doherty *et al.*, 2004; Cauda *et al.*, 2011; Dalley *et al.*, 2011). Definitively establishing a critical role, however, requires the combination of strong inferential power—testing ‘necessity’ by examining the consequences of focal interference—with transient functional modulation of the region, in the context of fully voluntary action. The temporal dynamics of the nucleus accumbens contribution to decision-making—within an unadapted system and an ecologically valid human environment—can thereby be established. Here we use therapeutic deep brain stimulation in patients with treatment-resistant psychiatric disorders to test the strong hypothesis that the human nucleus accumbens is critically involved in decision-making under risk, and further that this role is executed on-line, dynamically responsive to the behavioural context.

## Materials and methods

**Figure 1:**
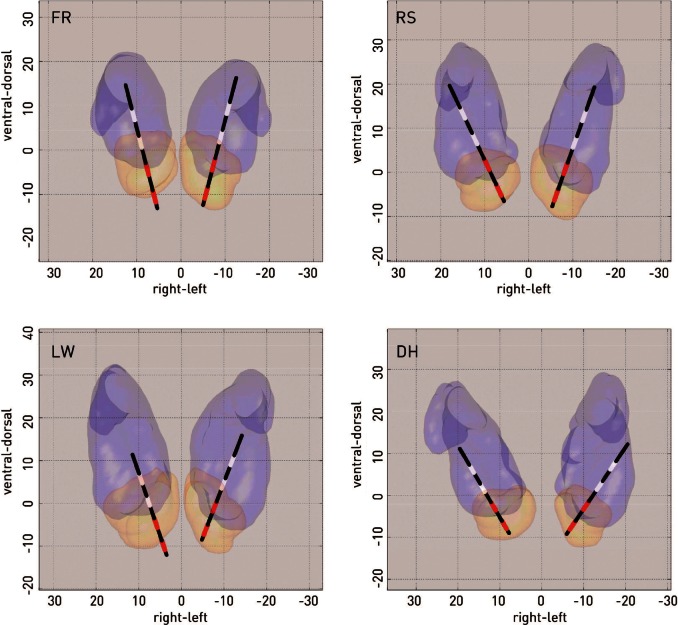
**Electrode locations**. Coronal view showing the critical electrode contacts (in red) in relation to the nucleus accumbens (translucent yellow), and the caudate (translucent blue), with dimensions relative to the anterior commissure. Note the distal two contacts used for nucleus accumbens stimulation are comfortably within target in each participant. The two proximal contacts (in white) were used for caudate control stimulation in Patients LW and DH. See Supplementary material for details.

We studied four patients (Supplementary Table 1) with bilateral, freshly-implanted deep brain electrodes sited in the region of the nucleus accumbens with the aid of a preoperative magnetic resonance scan fused with stereotactic frame-based CT imaging using standard clinical procedures (Fig. 1 and Supplementary material). Although the indication for therapy was psychiatric—drug-resistant obsessive compulsive disorder in three patients and major depressive disorder in one patient—their neuropsychology on most established clinical tests was within the normal range (Supplementary Table 2). The two most distal contacts of each electrode were positioned within the posterior part of the nucleus accumbens. The patients were studied 5 days after electrode implantation, during the usual evaluative period before permanent implantation of the stimulus device. None had received sustained stimulation through the electrodes prior to the experiment: all were therefore naïve and unadapted, both psychologically and physiologically. The participants were unselected except for ability and willingness to perform the experimental task. For behavioural reference, 17 healthy control participants also performed the task following the same protocol, in the absence of any implantation or stimulation.

**Figure 2:**
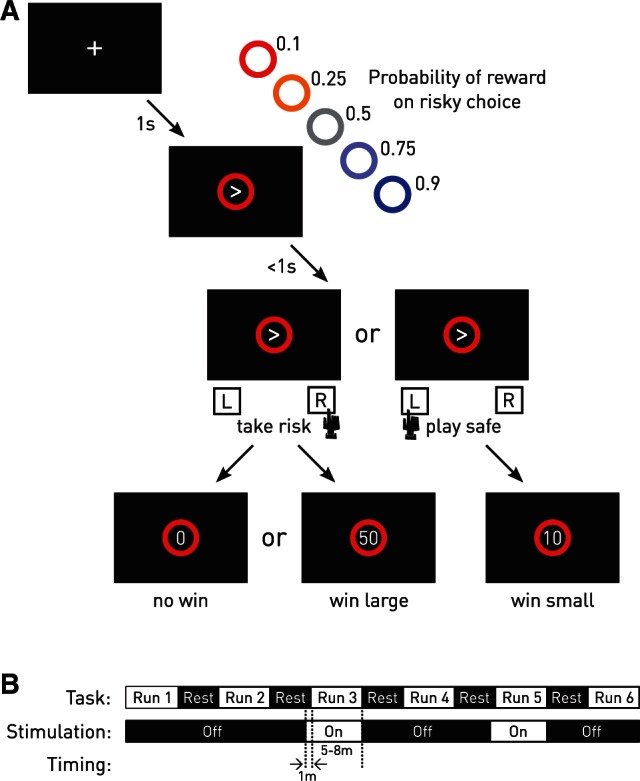
**Experimental design.** (**A**) Risky decision-making task. On each trial, the participants were forced to decide with a manual button press between taking an uncertain, risky, large reward option (follow the arrow) or a certain, safe, small reward option (go against the arrow) within 1 s of cue presentation. The probability of success of the uncertain option varied from 0.1 to 0.9, indexed by the colour of a ring around the arrow cue. Participants were not told the actual probabilities, only their relative order as suggested by the heat map, but learned by feedback during a block in advance of the experiment. The outcome was indicated with a number immediately after the response corresponding to points accrued towards the maximum total win of €30 across the whole experiment. The outcome on risky choices was either 0 or 50 points; the outcome on certain choices was always 10 points. The actual monetary reward was given at the end of the experiment. (**B**) Nucleus accumbens stimulation timings. The trials were arranged in six blocks of 160, with stimulation delivered—blind to the participant—during block 3 and 5, starting 1 min before the beginning of the block. See the online Supplementary material for details.

To capture decision-making under risk with high sensitivity to intraindividual changes over short time scales, we created a time-pressured, visually-driven behavioural task involving a two-alternative forced manual choice in the context of manipulated, probabilistic monetary reward (Fig. 2A and Supplementary material). On each of 960 trials, divided into six blocks (Fig. 2B), the participant was asked to choose between an uncertain, ‘risky’, large reward and a certain, ‘safe’, small reward within 1 s of the presentation of a visual cue. This cue indexed the probability of reward by its colour, in the range of 0.1, 0.3, 0.5, 0.7, and 0.9. The outcome of each choice—large reward, no reward, or small reward—was fed back to the participant immediately after the response, in the form of a numerical display of points won, translated at the end of the experiment into money up to a maximum amount of €30 across the entire experiment. Omissions (rare at a mean of 3.3% of all trials) were unrewarded. The association between the colour and the probability of reward was not given explicitly beyond the direction of increasing risk (from blue to red) but, rather, established by feedback learning during a practice block before the experiment proper began so as not to bias responding by *a priori* notions about the set probabilities.

**Figure 3:**
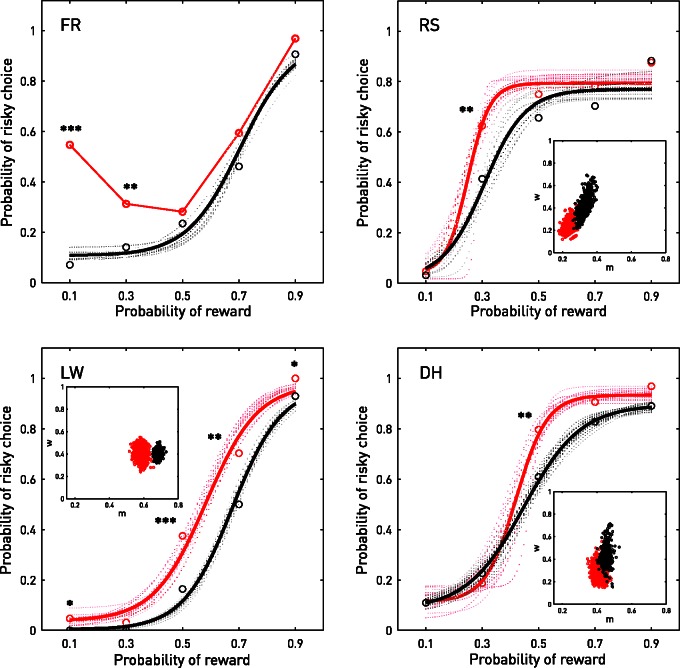
**Behaviour**. The relation between the probability of reward on risky trials and the propensity to choose them was modelled individually for each participant and for each condition (on and off stimulation) as a psychometric function of logistic form, estimated within a Bayesian framework with the aid of MCMC sampling. These estimates are line-plotted—in black for off and red for on—together with 20 random illustrative samples from the posterior distribution of functions (in saturation of the same colour proportional to the deviance). The ‘on’ condition in Patient FR produced non-monotonic behaviour that could not be modelled. The circles, analogously colour-coded, show the actual choice performance at each probability, with the threshold of significance indicated by asterisks (*P < 0.05, **P < 0.01, ***P < 0.001) as derived from a Fisher’s exact test with a two-tailed *P* estimated by the mid-*P* method. The inset sub-axes show the MCMC samples from the posterior distributions for the parameters of slope (*w*) and threshold (*m*) from which the functions are estimated. Note that stimulation produced a change in the thresholds for each participant, consistently in the direction of greater risk-seeking behaviour. See Supplementary material.

To parameterize risky decision-making we constructed a psychometric function of logistic form relating the probability of reward on the risky, potentially large reward option to the probability of choosing it over the safe, small reward option (Fig. 3 and Supplementary materials). The parameters were individually estimated within a Bayesian inferential framework with Markov Chain Monte Carlo (MCMC) sampling of the posteriors. Only four estimated parameters—threshold, slope, floor, and ceiling—thereby allowed us fully to characterize the participant’s behaviour and any change in response to nucleus accumbens stimulation. The threshold indexes the reward probability level above which the participant will tend to choose the risky option: a measure of risk-seeking or aversion. The slope indexes the variation in the tendency to choose the risky option with the probability of reward: a measure of risk sensitivity or indifference. The floor and ceiling parameters index the tendency to choose the risky or safe options respectively, regardless of reward probability: measures of reward type bias independent of the probability of reward.

To investigate the dynamic effects of electrical stimulation of the nucleus accumbens within this framework we examined performance during alternate short blocks without (‘off’) or with (‘on’) stimulation (3.5 V; 130 Hz frequency; 60 µs pulse-width), delivered simultaneously through both electrodes, blind to the patient, within a run of six blocks in total (Fig. 2B). To limit local current spread, we applied bipolar stimulation between the two nucleus accumbens contacts (with the most distal as cathode). The stimulation parameters corresponded to those commonly used in the therapeutic setting (Sturm *et al.*, 2003; Benabid *et al.*, 2009).

## Results

In the absence of stimulation, all patients exhibited a strong monotonic relation between choosing the risky option and the associated reward probability that rose from a low floor to a high ceiling, indicating good sensitivity to risk and little baseline bias for either option (Fig. 3, black curves). There was a mild tendency to risk aversion compared with controls, as previously described (Supplementary Fig. 1) (Smoski *et al.*, 2008; Admon *et al.*, 2012). Nucleus accumbens stimulation, however, induced a marked change in the decision behaviour of each participant manifesting as a consistent dynamic shift towards greater risk-seeking during the ‘on’ blocks (Fig. 3, red curves). This was manifest in significantly altered performance on at least one risk level for all four patients (see Fig. 3 for *P*-values). This difference was reflected in a consistent left shift in the posterior distributions of thresholds of the psychometric functions where they could be fitted (Fig. 3, insets). In Patient FR, the relation between risk and choice ceased to be monotonic, now exhibiting enhanced preference for very low probability risky rewards as well as an apparent shift of the function overall. With the exception of Patient FR, who reported mild elation in the first block of stimulation only, these dynamic changes were unaccompanied by any patient-reported correlate. On direct questioning after the experiment only Patient FR was able to correctly report one of the two blocks in which stimulation was delivered and no participant indicated any deliberate change in playing strategy at any stage of the experimental run. Stimulation did not significantly alter mean reaction times in Patient FR (on 592 ms, off 613 ms; *P* = 0.43) or Patient LW (on 710 ms, off 725 ms; *P* = 0.44), but produced a small but significant slowing in Patient RS (on 678 ms, off 650 ms; *P* = 0.012) and Patient DH (on 798 ms, off 765 ms; *P* = 0.0034), the reaction times being modelled with an ANOVA incorporating stimulation, choice, and reward probability as factors, including their interactions (Supplementary material). No other significant main effects or interactions were observed.

To confirm the anatomical-specificity of the observed nucleus accumbens stimulation effects, we performed a further stimulation session in two participants (Patients LW and DH) on a different day, in counterbalanced order (stimulator set to off during the intervening day). Experimental parameters were exactly the same except that bipolar stimulation was applied through the two most proximal electrode contacts (Fig. 1 in white), falling wholly within the caudate nucleus (again with more distal contact as cathode). No significant effect of stimulation on the decision function was observed in either participant (Supplementary Fig. 2), consistent with a nucleus accumbens-specific stimulation effect.

**Figure 4:**
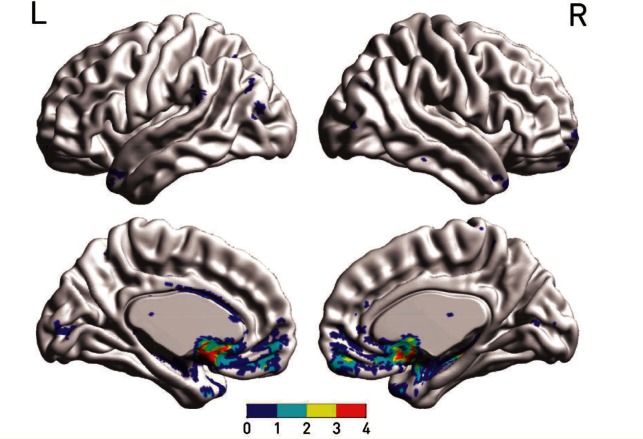
**Estimated cortical projections of the stimulated sites**. The overlay colours index the overlap between individual thresholded projections from the two stimulated sites in each participant, shown for medial and lateral surfaces of each hemisphere, as determined by preoperative diffusion tensor tractography seeded from the target following registration with the postoperative scan. Note the close similarity across participants, and overall pattern consistent with the predicted connectivity of nucleus accumbens. See Supplementary Fig. 3 for a comparison with caudate connectivity estimates. See Supplementary material for details.

In keeping with such anatomical specificity, maps of the cortical connectivity of the stimulated region within the nucleus accumbens (Fig. 4)—determined by seeding from that region within a whole-brain tractography analysis performed on the individual patient’s preoperative diffusion weighted imaging scans—closely paralleled the ventromedial prefrontal areas identified in human lesion studies of risky decision-making. (Supplementary material) (Clark *et al.*, 2008). They furthermore differed as expected from an identical analysis seeding from the caudate contacts (Supplementary Fig. 3).

## Discussion

These results overall indicate a significant stimulation effect of increased risk-seeking, in the context of maintained sensitivity to risk. They are consistent with a dynamic, on-line role of the nucleus accumbens in determining the contribution of risk to reward-seeking behaviour, establishing with an interventional method observed functional imaging correlates (Matthews *et al.*, 2004; Kuhnen and Knutson, 2005; Preuschoff *et al.*, 2006), and neurophysiology (Cohen *et al.*, 2008; Patel *et al.*, 2012). The effects were significant within-subject, across a narrow temporal scale, indicating a contemporaneous role in the absence of the possibility for any long term neural adaptation. That the participants were largely unable to report the stimulation argues against a global effect consequent on the participant’s own perception of any change in behaviour, or other psychologically complex reactions. Equally, the absence of a significant reduction in reaction time is inconsistent with accelerated responding heedless of risk, where in any event we would expect a flattening of the psychometric function and no consistent shift in the location of the threshold across the group. Rather, the data constrain the plausible interpretations to a specific effect on risk-seeking in circumstances of uncertain reward.

Our study of dynamic, unadapted modulation of the nucleus accumbens within a voluntary and relatively constrained task reveals a contrast with permanent adapted lesions in lower animals within highly learnt tasks, where impairment of risk sensitivity has been reported (Cardinal and Howes, 2005). Clearly, the context of focal modulation—both temporal and behavioural—may influence its impact, necessitating direct study of the mechanisms in awake, behaving humans. Equally, the effect of electrical stimulation need not be univalent, perhaps differentially affecting local nucleus accumbens processing and remote cortical connectivity as others have suggested (Figee *et al.*, 2013).

Moreover, the far greater development of the cortical regions implicated in decision-making in primates may make the behaviour less dependent on phylogenetically older, subcortical regions, imposing cortical-driven greater ‘rationality’—here manifest as risk sensitivity—on less sophisticated, impulsive tendencies. As reflected in our tractographic analyses (Fig. 4), the nucleus accumbens is closely interconnected with ventromedial prefrontal areas associated with making decisions under risk (Clark *et al.*, 2008). Indeed, the implication of the ventral striatum in circumstances of systematically irrational risk-taking (Smith *et al.*, 2014) suggests it is plausibly an important driver of maladaptive impulses such as the all too common propensity to gamble in the setting of fixed poor odds. Competitive as well as collaborative cortical-subcortical interactions are consistent with the data here.

The focus of our study was the form and direction of the behavioural perturbation induced by transient nucleus accumbens stimulation, not the effect on optimizing behaviour so as to maximize the monetary return. The participants universally performed on the risk-averse side of optimality—as defined by the risk probability at which choosing the risky option maximized the monetary return across the experiment—but the change induced by the stimulation cannot have been an acceleration of a drift towards the optimal for it was dynamic, specific to the stimulated blocks, distributed over the course of the experiment. Indeed, such effects would be expected to reduce the size of the observed effect rather than spuriously create it, for an uncorrected linear drift in the baseline across the experiment would reduce our power to detect block-specific effects. Furthermore, partitioning the data from the control participants in the same way—treating blocks 3 and 5 as ‘on’ and the rest as ‘off’—reveals essentially identical functions across the group (Supplementary Fig. 1, control plots). Similarly, the observed effects are not plausibly explained by an increased tendency to follow the arrow regardless of risk, for that would predict changes in more parameters of the risk function than just threshold.

The illumination of fundamental brain mechanisms aside, our results show that therapeutic stimulation of the nucleus accumbens at conventional clinical settings, even over very short timescales, may significantly increase risk-seeking behaviour in ways that need not be reportable by the patient. Clearly, such interventions are only considered in patients where less invasive approaches have been unsuccessful, and where close behavioural monitoring is the norm. Nonetheless, it is an aspect of behaviour for which no sensitive monitoring tools currently exist and may be detected only when it has already had an undesirable impact on the patient’s life. The reversible nature of stimulation does helpfully mitigate this possibility, but it needs careful consideration as this therapy becomes more widely used. Conversely, our results are obtained from patients with psychiatric disease—inevitably so—who will differ from the normal population. Their generalizability to normal physiology cannot be presumed, though the clinical and behavioural parameters in relation to decision-making are broadly within the range of the normal population.

Taken together, our results provide evidence of the contemporaneous criticality of the nucleus accumbens to decision-making under risk, and the consequences to behaviour of its disruption in a therapeutic context, establishing a platform for further interventional exploration of the role of the region in health and disease.

## Supplementary Material

Supplementary Table 1
