# Supplementary material for: Dynamic risk control by human nucleus accumbens
Source: Brain. 2015 Oct 1;138(12):3496–502. doi: 10.1093/brain/awv285 (PMC4655342; doi:10.1093/brain/awv285)
Supplement: Supplementary Table 1 [file brain_awv285_index.html]

Supplementary Data | Brain

## Supplementary Data

files

**Files in this Data Supplement:**

- Supplementary Data - pdf file
- Supplementary Data - tif file
