# Supplementary material for: Dynamic risk control by human nucleus accumbens
Source: Brain. 2015 Oct 1;138(12):3496–502. doi: 10.1093/brain/awv285 (PMC4655342; doi:10.1093/brain/awv285)
Supplement: Supplementary Table 1 [file awv285_SupplementaryMaterialR1.pdf]

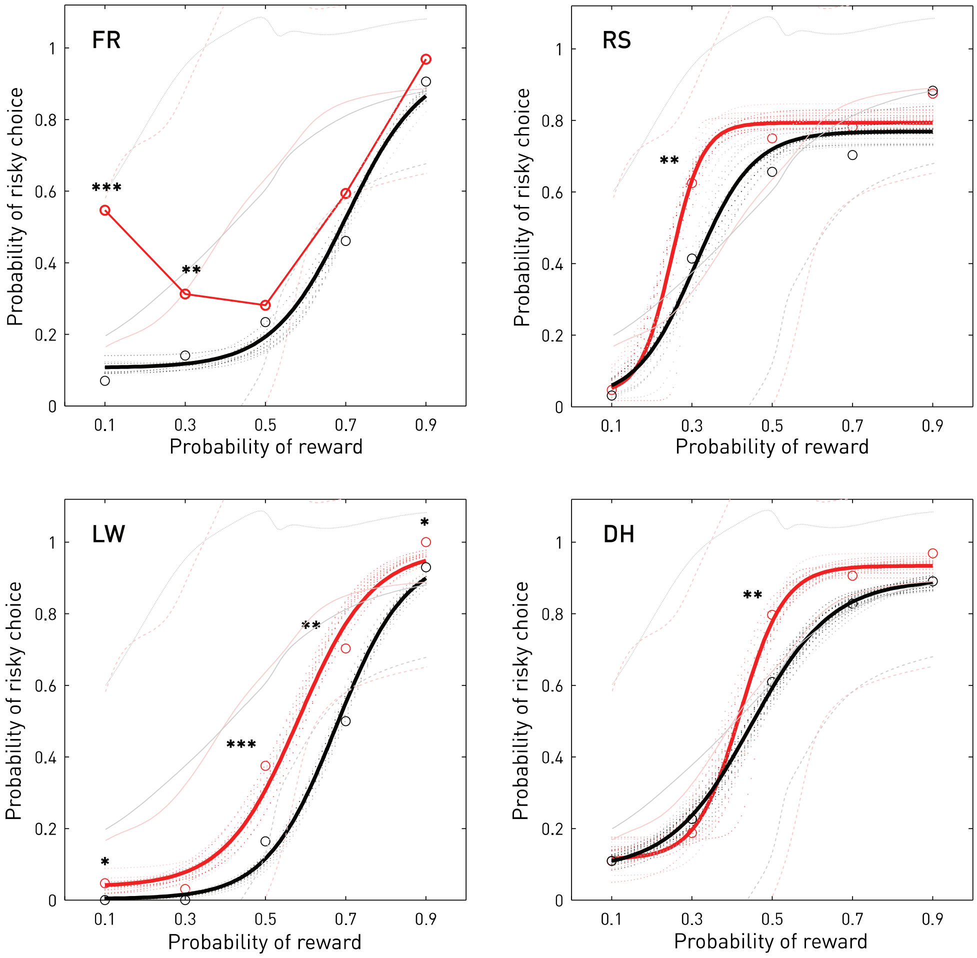

Figure S1. Patient data in the context of control performance. The psychometric functions depicted in Figure 3 in the paper are here reproduced against the distribution of performance across the 17 control participants. The patient plots are as before. The control participant plots show the group mean of the risk function evaluated across the interval 0.1 to 0.9 probability of reward (pale solid line), for the 1st, 2nd, 4th, and 6th blocks (in grey) and for the 3rd and 5th blocks (in pink). The dashed lines of the corresponding colour indicate + & - 2.5 standard deviations of the group distribution at each point of the function. This replicates the patient analysis where the two sets of blocks differ by stimulation, the factor of interest here. Note that there are no block effects of consequence and that patient baseline performance varies from normal to mildly risk averse relative to the population.

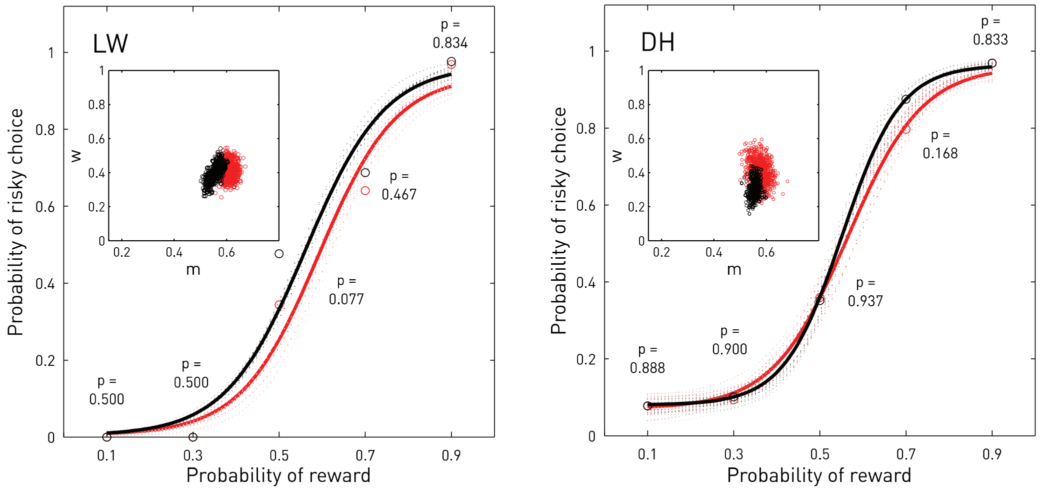

Figure S2. Behaviour during caudate stimulation. As before, the relation between the probability of reward following risky choices and the propensity to choose them was modelled individually for each participant and for each condition ('on' and 'off' stimulation) as a psychometric function of logistic form, estimated within a Bayesian framework with the aid of MCMC sampling. These estimates are line-plotted—in black for 'off' and red for 'on'—together with 20 random illustrative samples from the posterior distribution of functions (in saturation of the same colour proportional to the deviance). The circles, analogously colour-coded, show the actual choice performance at each probability, derived from a Fisher's exact test with a two-tailed p estimated by the mid-P method. The inset sub-axes show the MCMC samples from the posterior distributions for the parameters of slope (*w*) and threshold (*m*) from which the functions are estimated. Note that by contrast with the NAC, stimulation in the caudate produced no significant change in each participant.

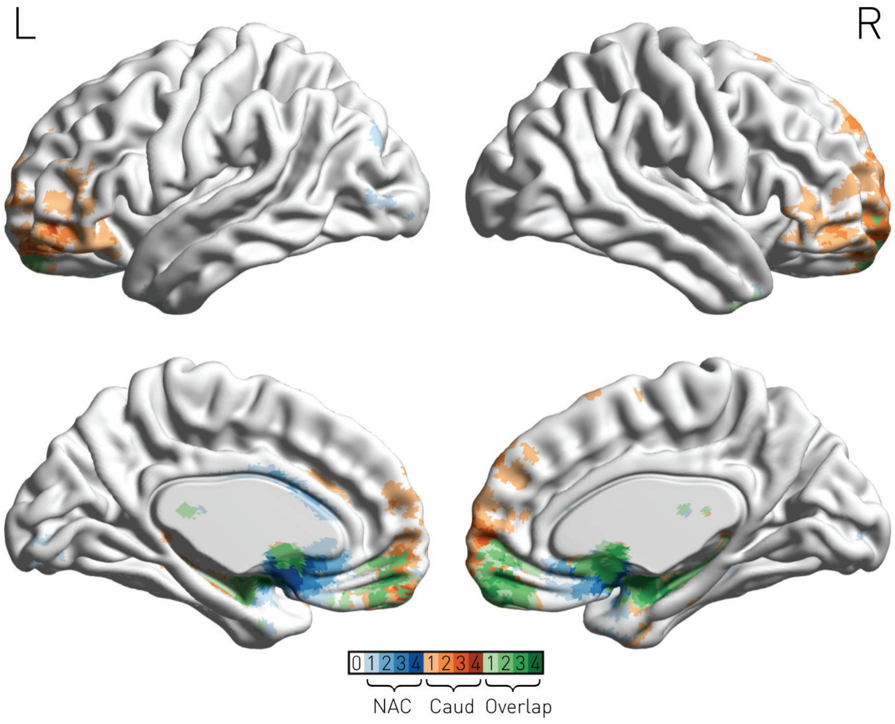

Figure S3. Visualization of connectivity differences between NAC and caudate stimulation sites. The colour overlays index the overlap (green hues) between individual thresholded projections from NAC (blue hues) and caudate (orange hues) sites in each participant, shown for medial and lateral surfaces of each hemisphere, as determined by pre-operative diffusion tensor tractography seeded from the target following registration with the post-operative scan. Note the clear, bilaterally consistent differences in cortical connectivity for the NAC and caudate sites, as predicted from the known connectivity of the targeted regions.

Table S1. Participant demographic and clinical characteristics.

Participant details	FR	RS	LW	DH
Age	28	28	45	40
Gender	F	M	M	F
Handedness	R	R-L	R	R
Schooling (years)	15	11	18	17
Age at onset	7	10	20	9
Duration (years)	21	18	25	31
Diagnosis	Obsessive Compulsive Disorder	Axis I: Obsessive Compulsive Disorder, Axis II: Schizoid Personality Disorder	Major Depressive Disorder	Obsessive Compulsive Disorder
SCID-I Coding	F42.8	F42.8	F33.2	F42.8
Comorbidity	None	None	Bipolar Disorder II	None
Obsessions	Aggressive, Contamination, Somatic, Miscellaneous	Aggressive, Sexual		Aggressive, Sexual, Religious, Miscellaneous
Compulsions	Cleaning, Washing, Miscellaneous	**		Cleaning, Repeating, Counting, Miscellaneous
Drug therapy (/day)	Setraline:200mg	Clomipramine:150mg<br>Sertraline:300mg<br>Oxitriptan:200mg	Lormetazepam:1mg<br>Methylphenidate:20mg<br>Risperidone:1mg<br>Valproate:500mg<br>Venlafaxine:75mg	Setraline:100mg<br>Pregabalin:300mg<br>Venlafaxine:150mg<br>Clorazepate dipotassium:30mg
Other previous brain stimulation therapies	Nil	Transcranial Magnetic Stimulation	Electroconvulsive Therapy	Nil
Yale–Brown Obsessive Compulsive Scale	31	13	2	33
Beck Depression Inventory	24	22	36	42
State-Trait Anxiety Inventory–State version	44	24	47	51
State-Trait Anxiety Inventory–Trait version	45	40	35	48
Clinical Global Impression-severity scale	5	5	5	6
Global Assessment of Functioning	25	25	40	30

** At disease onset, this patient presented with cleaning, checking and order compulsions, which responded to pharmacotherapy. His score on the Yale–Brown Obsessive Compulsive Scale is in the mild range, which reflects the relative insensitivity of this scale to primarily obsessive symptomatology.

Table S2. Patient neuropsychological data; raw scores (percentiles). Abbreviations, FCSRT: Free and Cue Selective Reminding Test; TMT: Trail Making Test (A and B); LNS: Letter Number Sequencing; CPT: Continuous Performance Test II; RT: Reaction time; WCST: Wisconsin Card Sorting Test; PE: Perseverative errors. Note that scores for the Stroop Test, pertain to the "colour-word page" interference subscale ("card C"). The performance is relative to standard reference intervals in the Spanish population.

Measure		FR	RS	LW	DH
FCSRT	Delayed Free Recall	13 (41-59)	6 (1)	10 (11-18)	13 (41-59)
	Total Delayed Recall	16 (>99)	12 (2)	15 (19-28)	16 (>99)
Verbal Fluency	Animals	19 (11-18)	23 (41-59)	27 (72-81)	18 (11-18)
	Initial "P"	15 (29-40)	16 (41-59)	21 (72-81)	20 (72-81)
	Excluded "E"	14 (41-59)	17 (82-89)	18 (90-94)	12 (29-40)
Stroop		29 (2)	42 (19-28)	42 (19-28)	36 (6-10)
TMT	A	36 (11-18)	31 (29-40)	22 (72-81)	22 (60-71)
	B	225 (<1)	183 (<1)	52 (60-71)	72 (19-28)
LNS		13 (72-81)	10 (29-40)	14 (90-94)	7 (3-5)
CPT	Hit RT	338.4	416.5	459.5	488.3
	Commissions	11	6	3	6
	Perseverations	0	0	0	1
WCST	Categories completed	4 (11-16)	6 (>16)	6 (>16)	6 (>16)
	PE	26 (4)	6 (>99)	5 (39)	4 (55)
	Non PE	27 (7)	6 (84)	6 (39)	3 (84)

Supplementary Experimental Procedures

Participants

Three patients suffering from treatment refractory obsessive compulsive disorder and one patient from treatment refractory major depression (Table S1) took part in this study. They were unselected except for willingness to take part in the study and a demonstrable ability to perform the task. Their psychiatric state and neuropsychological function were assessed using standard instruments in routine clinical use (Tables S1 & S2).

Seventeen healthy control participants (8 female; age range 28-47 years; mean 36.4 years) were also recruited solely for establishing a broad behavioural baseline for performance on the task. No intervention of any kind was carried out on them. The criterion for participation was absence of significant neurological history or psychiatric morbidity, the latter assessed with the Yale-Brown Obsessive Compulsive scale (Y-BOCS), Beck's Depression Inventory (BDI). For Y-BOCS, mean score (standard deviation) [range from maximum to minimum] was 2.1 (1.1) [1-5] and for BDI 3.1 (2.4) [0-8]. Thus, scores for all control participants were below our exclusion criteria of Y-BOCS > 7 and BDI > 9.

All participants provided written informed consent. The study had full approval from the Hospital Clinico San Carlos ethics committee.

Neurosurgical procedure

Electrode implantation was performed according to standard stereotactic procedures, employing frame-based magnetic resonance imaging fused with frame-based CT imaging for target determination. All patients underwent bilateral implantation of 4 direct-contact electrodes (model 3391, Medtronic Inc., Minneapolis, Minnesota). The target for the nucleus accumbens was determined by measuring the distances from the AC-PC line and the mid-sagittal plane, using a Medtronic Stealth Station Treon navigation system (Medtronic Inc., Minneapolis, Minnesota). The target was placed close to the bed nucleus of the stria terminalis at the coordinates reported by Sturm et al.(Sturm *et al.*, 2003): 2.5 mm rostral to the anterior border of the anterior commissure, 4 mm ventral to the AC-PC line, and 7 mm lateral to the mid-sagittal plane. A trajectory was planned to reach the target and for placing the rest of the contacts (four 3.0-mm contacts in total, 4.0-mm spacing between contacts, 1.5-mm spacing after most distal contact) at several points along the striatum, avoiding the ventricles. The contacts are coded from 0 (ventral) to 3 (dorsal) and are independently programmable.

On the operative day, a burr hole was made on each side, the dura opened and a microelectrode FC 4000 (FHC, Bowdoin, Maine, USA) was advanced into the ventral striatum towards the target using a Microdrive (Medtronic NexDrive). In the case of relatively silent neuronal activity, the target was advanced 2 mm anteriorly. After microelectrode recording, the model 3391 macroelectrode was implanted at the determined target, and clinical effects were briefly tested using an external tester (Medtronic Model 3625 Test Stimulator). For each contact, monopolar stimulation at 3.5 V was delivered at 130 Hz (pulse width 60 μs), and then increased up to 5 V to screen for undesired side effects (none of which appeared in any case). Intraoperative X-ray was used to verify the positioning of the two electrodes. Post-operative CT and T1-weighted MRI verified final electrode position. After implantation, the lead extensions were externalized for 6 days.

Behavioural task

The task parameters were invariant across all participants. The participant was seated in front of a laptop with a 12 inch screen (diagonally) approximately 1 meter away, interacting with it through the two buttons of a mouse connected via a USB interface. The behavioural task was implemented in Presentation Version 14.9, Build 06.09.11 (http://www.neurobs.com/).

The experimental task was divided into 6 blocks of 160 trials each, collected during a single session. This session was immediately preceded by a single block of 160 trials introduced to familiarize the participant with the task and to learn the risk probabilities. Participants rested for 5 minutes between blocks. Participants LW and DH underwent a second session on a separate day in which the caudate rather than the NAC electrodes were used for the stimulation blocks. For participant LW, this session preceded the NAC session, counterbalancing for order effects across the two patients.

The task is schematized in Figure 2. The participants were told that they would be playing a simple computer game where they had to make rapid decisions about choosing between uncertain, large rewards and certain, small rewards, which would be translated at the end of the experiment to a maximum monetary reward of 30 euro in total, depending on the aggregate outcome of all trials. They were then taken through the details of an individual trial, and the decision they had to make on each trial.

Each trial began with a fixation cross at the centre of the screen. One second later, the cross was replaced by a white arrow cue pointing either left or right, surrounded by a ring of one of five colours: red, orange, grey, light blue, or dark blue. The

participant was instructed to respond to this cue within 1 second with a button press either corresponding to the direction of the arrow or against it. Following the direction of the arrow indicated choosing the uncertain, "risky" option, going against it indicated choosing the certain, "safe" option. The probability of reward on choosing the risky option was variable, indicated by the colour of the ring surrounding the arrow cue. The probabilities were set at red: 0.1, orange: 0.3, grey: 0.5, light blue: 0.7, and dark blue: 0.9. Rewarded trials in which the risky option was chosen earned the participant 50 points per trial, unrewarded risky option choice 0 points per trial. The probability of reward on choosing the safe option was fixed at 1.0, the reward being 10 points. Omissions were unrewarded. Equal numbers of each colour trial type were performed in each block, with their order randomized across the block. Feedback was given immediately after the response in the form of a number indicating the points won—50, 0, or 10—replacing the arrow cue for a duration of 1 second. Omissions were followed by the presentation of an '!' sign. The next trial began immediately thereafter.

Participants were not told the reward probabilities associated with each risk colour but they were told that they varied on the red/blue axis, red being the high risk end of the continuum and blue the low risk end. They were also exposed to the probabilities in the practice block before the experiment proper. All participants clearly differentiated between different levels of risk as this was unequivocally reflected in their behaviour as reported in the main text. At the conclusion of testing the participants were given the full amount of 30 euro regardless of their performance. None of the participants knew before (or during any part of) the experiment that the amount they were given at the end would be indifferent to their performance. At the time of their experiment, none of the participants had had any contact with any previous participant, as far as we are aware, and so would have had no source of information other than the experimenters.

Stimulation was delivered—blind to the participant—only during block 3 and 5, starting 1 minute before the beginning of the block (see Figure 2B). Naturally, only patients received stimulation of any kind. The control participants simply completed the same number of blocks in sequential order.

Electrode stimulation

Bilateral nucleus accumbens stimulation was delivered via a constant current stimulator as square pulses (Medtronic Model 3625 Test Stimulator). When the NAC was stimulated, bipolar stimulation at 3.5 V between the two most distal contacts (coloured red on Figure 2, negative polarity most distal) was delivered at 130 Hz (pulse width 60 μs). When the caudate was stimulated, the same parameters were

used except that the current was delivered through the two most proximal pair of electrode contacts (coloured white in Figure 2). In all cases, the bipolar voltage was ramped up from 0 to 3.5 V over 15 s, starting 1 minute before runs 3 and 5 in each session. Although the target location of the most distal electrode contact is NAC shell (Sturm *et al.*, 2003) given that we apply bipolar stimulation between this and the adjacent contact, we make no claims as to the anatomical NAC sub-region (shell vs. core) that is stimulated. The stimulation parameters were informed by those conventionally used in the therapeutic setting in subcortical regions (Benabid *et al.*, 2009; Sturm *et al.*, 2003).

The sequence of "on" and "off" blocks in each experiment was chosen so as to maximise the contrast between the two conditions at the single subject level given no prior knowledge of the duration of the behavioural effects of stimulation. Beginning with two "off" blocks ensured that a stable baseline could be established in the event of the "on" behavioural effect extending across the subsequent blocks. An alternating sequence of "on" and "off" was then terminated with an "off" block so as to capture two "on-off" and two "off-on" transitions in each patient, where each replication is distributed in time so as to offset any linear drift effects.

Behavioural analysis

Choice behaviour. Analysis of the psychometric function was done with the aid of the Matlab version of Psignifit 3.0ß (http://psignifit.sourceforge.net/). All analyses were done independently for each subject.

In brief, the relation between the probability of choosing the risky option and the associated probability of reward was modelled as the binomial mixture model conventionally used for psychophysical functions (Wichmann and Hill, 2001). The core of this was a logistic function with two parameters, m, and w, corresponding to the location and width of the function. The location is defined as the horizontal position of the sigmoid, halfway between the upper and lower asymptotes. The width is defined as the width of the interval in which the sigmoid rises rapidly, i.e. relatively close to the threshold, here in line with common practice chosen to be the interval 0.1 to 0.9. The model also incorporated parameters for the 'base rate', γ, the tendency for the participant to choose the risky option by default, even when the probability of success is very low; and the 'ceiling rate', λ, the tendency for the participant to choose the safe option even when the probability of success with the risky option is very high.

The model was constructed and estimated within a Bayesian inferential framework with uniform priors for each of the four parameters covering 0 to 1 for m, 0 to 10 for w, 0 to 0.5 for γ, and 0 to 0.5 for λ. Note that these ranges cover essentially the full

range of possibilities compatible with performing the task meaningfully and are well within the observed values. The posterior probabilities were estimated using Markov Chain Monte Carlo methods (MCMC) involving 4000 samples of which the final 2000 were used to estimate the parameter distributions. The uncertainty of the estimates was illustrated in Figure 3 by drawing, for each function, 20 random samples of the posterior distribution of functions and displaying them as dotted lines with colour saturation indexing the degree of deviance of each estimate. We also plot the MCMC samples of the posterior distributions for slope and threshold in the figure sub-axes so as to convey the full difference in the estimated parameters.

So as to set the performance of the patients against the background of the normal population we fitted functions identically to all control participants individually and evaluated them for every point within the interval 0.1 to 0.9 probability of reward. We then derived a mean function across all twenty control participants, together with a 2.5 standard deviation interval envelope for each tail of the distribution. The patient results in Figure 3 of the paper are replotted in Figure S1 with this control envelope in the background.

Independently of this parameterisation of the behavioural data, we conducted direct pairwise comparisons at each of the five fixed probabilities of reward for each patient. In this analysis, the number of trials on which the risky option was taken was compared between the 'on' and 'off' conditions, separately for each reward probability. The relatively conservative Fisher's exact test was used, with the two-tailed p value being estimated by the mid-P method.

Reaction time behavior. The effect of stimulation on reaction time was analysed with a 2x2x5 ANOVA with categorial factors stimulation ('on' vs 'off'), choice (risky vs safe), and probability of reward on the risky choice (0.1 to 0.9 in 5 steps). The choice factor was nested within the probability of reward factor. Main effects and interactions were modelled. As before, all analyses were done independently for each subject. Reaction times faster than 200ms were excluded from analysis as anticipations. These analyses were done in Matlab using the *anovan* function.

Imaging

Probabilistic Tractography: Imaging

A 3T Siemens TRIO system was used to acquire MPRAGE T1-weighted anatomical images with 1mm^3 resolution (repetition time (TR), 2300 ms; echo time (TE), 2.98 ms; flip angle, 9°). Acquisition of echo planar diffusion-weighted images was based on parameters used in previous probabilistic tractography studies of the basal ganglia (Draganski *et al.*, 2008). Each volume consisted of 40 axial slices of 2.3mm thickness

with no interslice gaps and an acquisition matrix of 96 x 96 in a field of view (FoV) of 220 x 100 mm, resulting in 2.3 x 2.3 x 2.3 mm^3 isotropic voxels (TR, 5800 ms; TE, 103 ms; flip angle, 90°; bandwidth, 2004 Hz/pixel). In order to increase the SNR, we acquired two contiguous sequences of 128 diffusion-weighted images. Each dataset consisted of 64 images with diffusion gradients applied along 64 non-collinear encoding directions for two different diffusion sensitization strengths (*b* = 500, 1000 s/mm2), and one additional image with no diffusion weighting (*b* = 0 s/mm2). A *b*-value of zero delivers a T2-weighted EPI image for anatomical reference.

A 1.5T GE MR system was used to acquire post-operative T1-weighted images. We employed a SPGR sequence to obtain high-resolution anatomical images with 1mm^3 resolution (TR, 10.9 ms; TE, 2.14 ms; flip angle, 15°). Post-operative computed tomography (CT) images with (0.5x0.5x1.25mm) resolution were acquired in a CT Philips Brilliance 64-channels system.

Probabilistic Tractography: T1 and CT pre-processing to map electrode's contacts position. Diffusion-weighted images were pre-processed using FSL 5.0.6 (http://fsl.fmrib.ox.ac.uk/fsl/fslwiki/). Non-brain tissue was removed from the pre-operative T1-weighted images using FSL-BET. Post-operative CT images were thresholded to obtain skull masks in order to remove bone from the image. Skull stripped CT images were co-registered to the pre-operative T1 image using FSL-FLIRT affine linear transformation. Post-operative CT images were again thresholded at an intensity of 1500 Hounsfield units to retain just the electrodes artefacts in the same orientation as the T1 image. The positions of the extreme of the lowest/highest tip of the most ventral/dorsal contacts were visually identified. In one case it was necessary to overlay the post-operative T1-weighted images for a correct estimation of the contacts position. We computed the coordinates of the centroid of the contacts using trigonometric functions. A volume of activated tissue (VAT) was defined by linearly scaling up ellipsoids centred in the contact centroids according to a DBS spatial activation spread model (Chaturvedi *et al.*, 2013). The original size of the ellipsoids was *a*=1.93mm; *c*=1.50mm for the deepest NAC and the deepest caudate electrode, and *a*=1.63mm; *c*=1.20mm for the second NAC and second caudate electrodes, where *a* is the perpendicular radius to the electrode, and *c* is the transversal radius.

Probabilistic Tractography: Anatomical Connectivity. DWI data were pre-processed using FSL-BET for non-brain tissue removal, and FSL-FDT for eddy currents correction. Estimation of the diffusion parameters was performed following a Bayesian approach (Behrens *et al.*, 2003), using a multi-shell model for the fitting of the parameters (Jbabdi *et al.*, 2012). White matter connectivity was quantified using probabilistic tractography with FSL-Probtrackx (http://fsl.fmrib.ox.ac.uk/fsl/fsl-4.1.9/fdt/fdt_probtrackx.html). The previously defined VAT for both left and right

hemisphere electrodes composed the seed mask for the tractography analyses, separately for the NAC and caudate electrodes. 5000 pathways per voxel in the seed ROIs were drawn with a maximum length of 2000 steps and a step-length of 0.5mm. Pathways with steps in which a sharp angle of 30° or higher occurred were discarded. We next extracted subject-specific cortical masks using Freesurfer 5.1.0, and these cortical masks were used to restrict the pathways in the tractography map, i.e. only those tracts passing through the cortical mask were considered. The b0 images were co-registered to the pre-operative T1 image using FSL-FLIRT affine linear transformation, and later normalized, using non-linear transformation FSL-FNIRT to $1mm^3$ T1 MNI template. These transformations were concatenated and applied to the tractography maps. The tractography maps were transformed to probabilities, dividing by the total number of pathways in the map. Then the maps were binarised, set at 1 for all values greater than zero, and summed across subjects in order to have a representation of the frequency in which a cortical voxel is reached across the group of 4 participants. These frequency maps are represented in a cortical surface in Figure 4 for the NAC, and in Figure S3 for NAC and caudate combined, showing the extent of overlap.

References

Behrens TEJ, Woolrich MW, Jenkinson M, Johansen-Berg H, Nunes RG, Clare S, et al. Characterization and propagation of uncertainty in diffusion-weighted MR imaging. Magn Reson Med 2003; 50: 1077–1088.

Benabid AL, Chabardes S, Mitrofanis J, Pollak P. Deep brain stimulation of the subthalamic nucleus for the treatment of Parkinson's disease. The Lancet Neurology 2009; 8: 67–81.

Chaturvedi A, Luján JL, McIntyre CC. Artificial neural network based characterization of the volume of tissue activated during deep brain stimulation. J Neural Eng 2013; 10: 056023.

Draganski B, Kherif F, Klöppel S, Cook PA, Alexander DC, Parker GJM, et al. Evidence for Segregated and Integrative Connectivity Patterns in the Human Basal Ganglia. J. Neurosci. 2008; 28: 7143–7152.

Jbabdi S, Sotiropoulos SN, Savio AM, Graña M, Behrens TEJ. Model-based analysis of multishell diffusion MR data for tractography: How to get over fitting problems. Magn Reson Med 2012; 68: 1846–1855.

Sturm V, Lenartz D, Koulousakis A, Treuer H, Herholz K, Klein JC, et al. The nucleus accumbens: a target for deep brain stimulation in obsessive–compulsive- and anxiety-disorders. Journal of Chemical Neuroanatomy 2003; 26: 293–299.

Wichmann FA, Hill NJ. The psychometric function: I. Fitting, sampling, and goodness of fit. Perception & Psychophysics 2001; 63: 1293–1313.
