# Supplementary figures and images for: Dynamic risk control by human nucleus accumbens
Source: Brain. 2015 Oct 1;138(12):3496–502. doi: 10.1093/brain/awv285 (PMC4655342; doi:10.1093/brain/awv285)

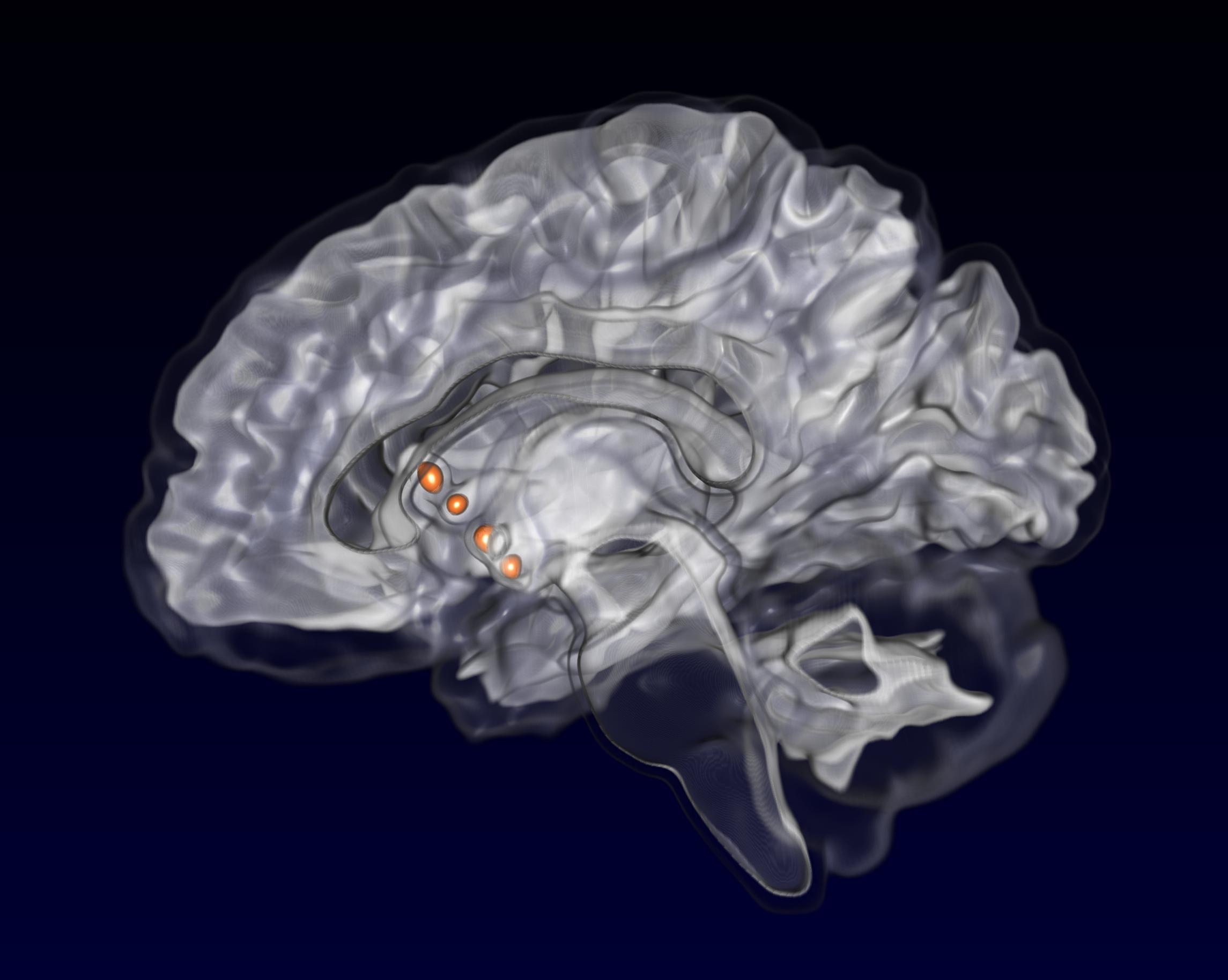

Supplement: Supplementary Table 1 [file ebfefc3379f0bd1b2ffba457578ded50_brain-2015-01141-File007.tif]
